# An experimental strategy to probe Gq contribution to signal transduction in living cells

**DOI:** 10.1016/j.jbc.2021.100472

**Published:** 2021-02-25

**Authors:** Julian Patt, Judith Alenfelder, Eva Marie Pfeil, Jan Hendrik Voss, Nicole Merten, Funda Eryilmaz, Nina Heycke, Uli Rick, Asuka Inoue, Stefan Kehraus, Xavier Deupi, Christa E. Müller, Gabriele M. König, Max Crüsemann, Evi Kostenis

**Affiliations:** 1 Molecular, Cellular and Pharmacobiology Section, Institute for Pharmaceutical Biology, University of Bonn, Bonn, Germany; 2 PharmaCenter Bonn, Pharmaceutical Institute, Pharmaceutical and Medicinal Chemistry, University of Bonn, Bonn, Germany; 3 Graduate School of Pharmaceutical Sciences, Tohoku University, Sendai, Miyagi, Japan; 4 Institute for Pharmaceutical Biology, University of Bonn, Bonn, Germany; 5 Laboratory of Biomolecular Research and Condensed Matter Theory Group, Paul Scherrer Institute, Villigen, Switzerland

**Keywords:** loss-of-function mutagenesis, Gαq, G protein inhibitor, FR900359, UBO-QIC, YM-254890, heterotrimeric G protein, dynamic mass redistribution (DMR), label-free biosensor, AC, adenylyl cyclase, Arg60, arginine 60, CCh, carbachol, DMR, dynamic mass redistribution, FCS, fetal calf serum, FR, FR900359, G proteins, guanine nucleotide-binding proteins, GEF, guanine nucleotide exchange factor, GPCR, G protein–coupled receptor, HA, hemagglutinin, HBSS, Hanks' buffered salt solution, HTRF, homogeneous time resolved fluorescence, IP1, inositol monophosphate, PDB, Protein Data Bank, PLC, phospholipase C, PTX, pertussis toxin, RTK, receptor tyrosine kinases, SRE, serum response element, YM, YM-254890

## Abstract

Heterotrimeric G protein subunits Gαq and Gα11 are inhibited by two cyclic depsipeptides, FR900359 (FR) and YM-254890 (YM), both of which are being used widely to implicate Gq/11 proteins in the regulation of diverse biological processes. An emerging major research question therefore is whether the cellular effects of both inhibitors are on-target, that is, mediated *via* specific inhibition of Gq/11 proteins, or off-target, that is, the result of nonspecific interactions with other proteins. Here we introduce a versatile experimental strategy to discriminate between these possibilities. We developed a Gαq variant with preserved catalytic activity, but refractory to FR/YM inhibition. A minimum of two amino acid changes were required and sufficient to achieve complete inhibitor resistance. We characterized the novel mutant in HEK293 cells depleted by CRISPR–Cas9 of endogenous Gαq and Gα11 to ensure precise control over the Gα-dependent cellular signaling route. Using a battery of cellular outcomes with known and concealed Gq contribution, we found that FR/YM specifically inhibited cellular signals after Gαq introduction *via* transient transfection. Conversely, both inhibitors were inert across all assays in cells expressing the drug-resistant variant. These findings eliminate the possibility that inhibition of non-Gq proteins contributes to the cellular effects of the two depsipeptides. We conclude that combined application of FR or YM along with the drug-resistant Gαq variant is a powerful *in vitro* strategy to discern on-target Gq against off-target non-Gq action. Consequently, it should be of high value for uncovering Gq input to complex biological processes with high accuracy and the requisite specificity.

Heterotrimeric αβγ guanine nucleotide-binding proteins (G proteins) are the main transducers of G protein–coupled receptors (GPCRs), the largest family of membrane proteins in mammalian cells (1, 2, 3, 4, 5, 6). G proteins mainly relay chemical and physical information from GPCRs to the cell interior to regulate numerous intracellular responses. In this manner, the GPCR–G protein–signaling cascade contributes to an amazing repertoire of physiological and pathophysiological events that are relevant for the regulation of blood pressure, cell proliferation, and metabolism, among many other vital functions (7, 8, 9, 10). At the molecular level, ligand-activated GPCRs act as guanine nucleotide exchange factors (GEFs) to stimulate GDP/GTP exchange on the G protein α subunit. This is followed by heterotrimer dissociation into Gα and Gβγ subunit complexes, which subsequently interact with their downstream effectors (5, 6, 10, 11, 12, 13).

Despite their central role in GPCR-mediated signal transduction, only a handful of pharmacological agents are available for specific disruption of G protein signaling (14, 15, 16, 17, 18). Of the four major G protein families (Gi/o, Gs, Gq/11, and G12/13), Gi/o proteins are effectively hindered from signal transmission by pertussis toxin (PTX) (19), whereas cholera toxin only masks Gs signaling by persistent activation of the Gs-adenylyl cyclase (AC) cascade (20). Unlike these two bacterial toxins, which act *via* covalent modification of their cognate Gαi and Gαs subunits, respectively (21), Gq inhibitors FR900359 (FR) and YM-254890 (YM), two naturally occurring cyclic peptides (22, 23), belong to a distinct yet particularly attractive subgroup of noncovalent cell-permeable signaling inhibitors. Mechanistically, both depsipeptides silence function of the Gq family members Gq, G11, and G14 *via* inhibition of GDP release, the rate-limiting step in G protein activation (24, 25). Thereby, FR and YM lock their cognate Gαβγ heterotrimers in the inactive GDP-bound form. Thus far, both depsipeptides are considered potent and highly selective for Gq over all other Gα families (24, 25, 26, 27, 28), explaining their widespread use to explore the biological consequences arising from specific Gq inhibition *in vitro*, *ex vivo*, and *in vivo* (14, 22, 29, 30, 31, 32, 33, 34, 35, 36, 37, 38, 39, 40, 41, 42, 43, 44, 45, 46, 47, 48, 49, 50, 51, 52, 53, 54, 55, 56, 57, 58, 59, 60).

Despite prior in-depth characterization of FR and YM (22, 24, 25, 26, 27, 28, 60, 61, 62, 63), two recent investigations have cast doubt on the selectivity profiles of both inhibitors. Although one study claimed FR to also inhibit Gβγ-mediated Ca^2+^ signaling downstream of Gi-coupled GPCRs (64), a recent report suggested YM to act as a broad-spectrum inhibitor for Gq and Gs proteins, and, additionally, as biased Gi inhibitor (65). Thus, an important emerging research question is, whether FR and YM exert such nonselective effects by directly inhibiting G proteins other than Gq, G11, and G14 or by inhibiting only its canonical targets in signaling networks that cooperate with other G protein classes to control a given effector system. The latter possibility is suggested by a large body of experimental evidence showing that GPCRs interact with multiple G proteins to cooperatively regulate downstream effectors. For example, certain AC isoforms are activated synergistically by Gαs and Ca^2+^ evoked by Gq/11, whereas others are activated by Gαs and Gβγ released from Gi/o heterotrimers (66, 67). Moreover, phospholipase C (PLC) β2 and β3 isoforms are synergistically activated by Gαq and Gβγ, the latter originating from Gi/o heterotrimers (68, 69, 70, 71, 72). For the above reasons, clarification of the purported nonselective pharmacology of FR and YM is important for basic science where both inhibitors are used to probe the function of Gq/11 (14, 29, 30, 31, 32, 33, 34, 35, 36, 37, 38, 39, 40, 41, 42, 43, 44, 45, 46, 47, 48, 49, 50, 51), as well as for translational science where both are explored as therapeutic leads in asthma (52), ocular melanoma (53, 54, 57, 58, 59), and cardiovascular diseases (22, 55, 56, 60).

To unambiguously demonstrate causality of FR (and YM) effects *in vitro*, a Gαq variant is needed that closely resembles the signaling phenotype of the WT protein, while being completely refractory to inhibitor action. Herein, we report design, identification, and functional characterization of a Gαq variant that fulfills this demand. Using HEK293 cells depleted by CRISPR-Cas9 of Gαq and Gα11 (hereafter HEK-ΔGq/11), we found that a minimum of two amino acid replacements was required and sufficient to completely eliminate FR binding to and inhibition of Gq^WT^, while maintaining Gq catalytic function. We then took advantage of this drug-resistant Gαq variant and used it, head-to-head, with Gαq^WT^ in a battery of functional assays, to unveil that (i) Gq inhibitors FR and YM exert their cellular effects by specific targeting of Gq family proteins and (ii) their combined application with a drug-resistant Gαq variant constitutes a powerful experimental approach to discern on-target Gq from off-target non-Gq action. We envisage that the here-described G protein-ligand system holds great promise to be applied to virtually any cell type to decode Gq contribution within signaling networks that are controlled cooperatively by Gq/11 and other classes of G proteins.

## Results

### Engineering a fully functional but FR- and YM-resistant Gαq isoform

**Figure 1:**
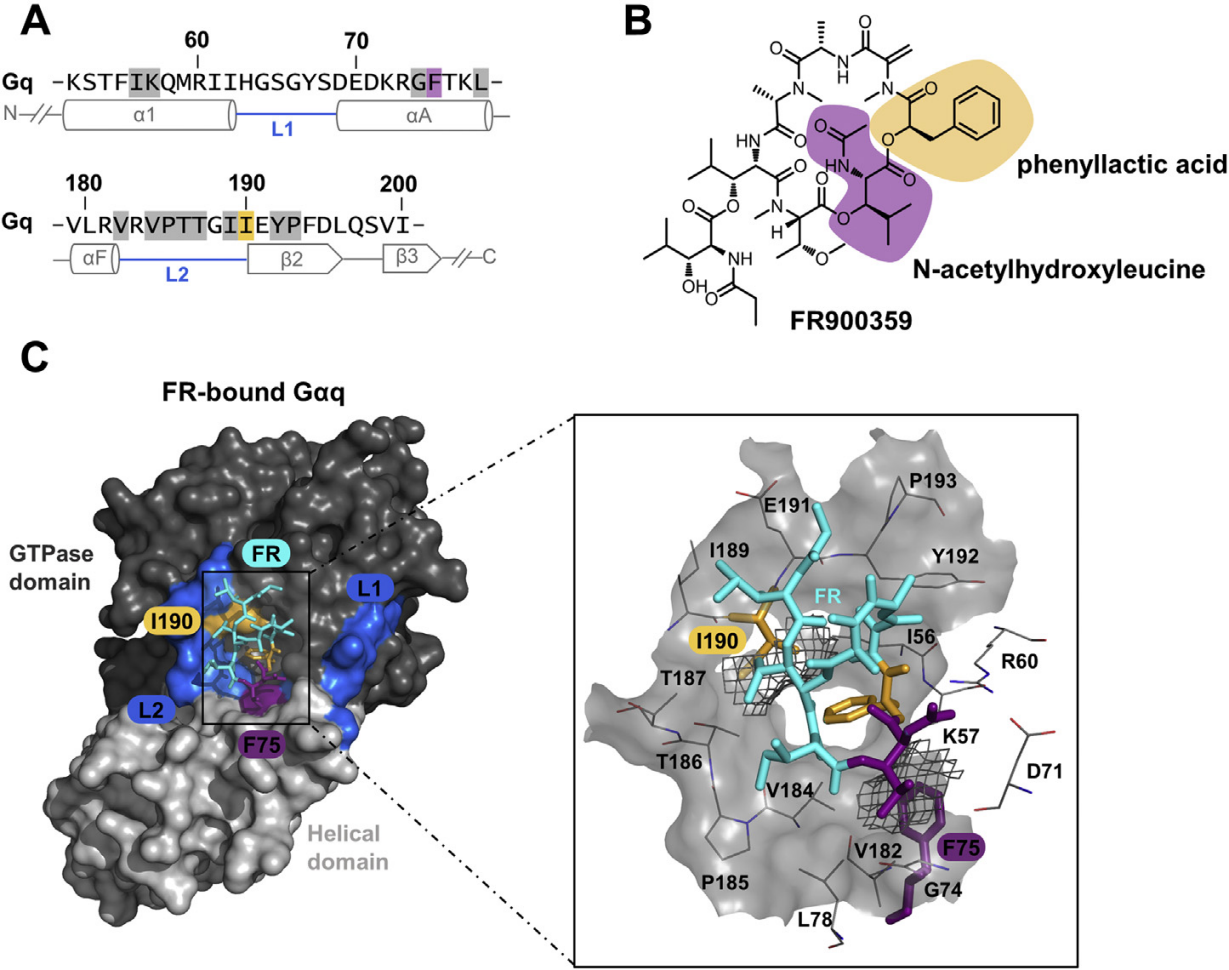
**Gαq residues phenylalanine 75 and isoleucine 190 form crucial hydrophobic interactions with FR**. *A*, amino acid sequence and secondary structure features of Gαq. Secondary structure assignments (α-helices, β-strands, linker regions) and ruler numbering were derived from Gαi/q (PDB code: 3AH8). The amino acids Phe75 and Ile190, previously defined as important for FR-Gαq interaction, are highlighted in *violet* and *ocher*, respectively. Gαq residues constituting the key hydrophobic interaction network for FR and YM are *boxed* in *light gray*. *B*, the chemical structure of FR with indicated building blocks in *ocher* and *violet*, respectively. *C*, the surface representation and detailed view of FR-bound Gαi/q tertiary structure (PDB code: 3AH8) composed of the GTPase and helical domain that are connected by linker 1 (L1) and linker 2 (L2; =switch I), respectively. FR (*stick* representation) was modeled into the interdomain cleft assuming equivalent anchor points as compared with YM. Phe75 and Ile190 are shown as *stick* models (zoom-in view) in *ocher* and *violet*, respectively. The *gray mesh* illustrates the vdW surfaces of Gαq residues Phe75 and Ile190; *light gray transparent* surfaces feature the vdW surface of the entire hydrophobic binding pocket. Oxygen and nitrogen atoms are colored in *red* and *blue*, respectively. FR, FR900359; PDB, Protein Data Bank; vdW, van der Waals; YM, YM-254890.

**Figure 2:**
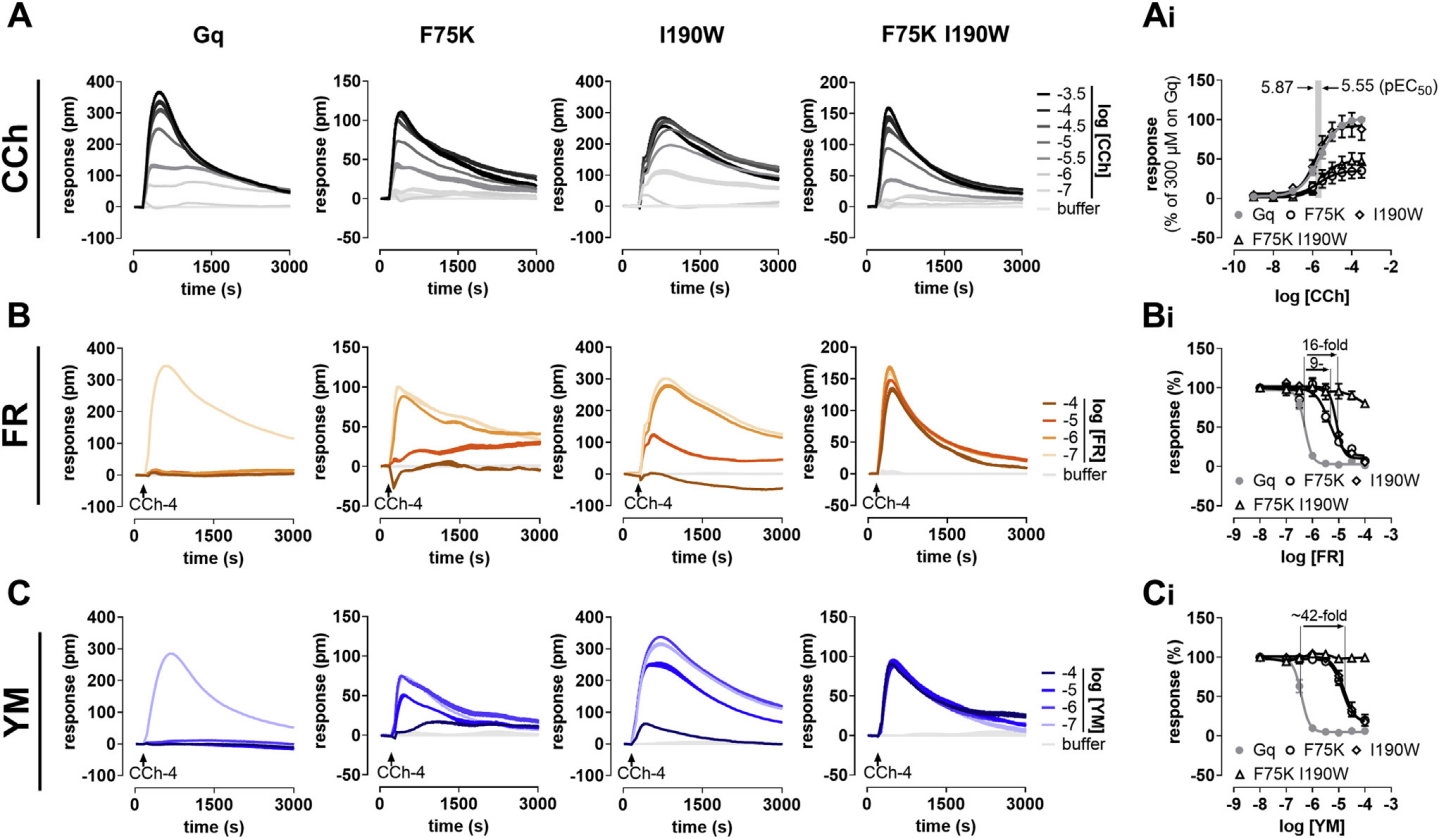
**Function and inhibition sensitivity toward FR and YM of Gαq single and double mutants**. *A*, concentration-dependent activation profiles of carbachol (CCh) in HEK-ΔGq/11 cells transiently transfected to express WT or mutant Gαq isoforms using label-free DMR biosensing. *A*_*i*_, concentration-effect curves of the traces depicted in (*A*), normalized to Gq^WT^ activation evoked with 300-μM CCh. *B*, *C*, concentration-dependent inhibition of cell responses by FR or YM using 100-μM CCh. *B*_*i*_, *C*_*i*,_ concentration-inhibition relationships for the traces shown in (*B* and *C*). DMR recordings are representative (mean + SE) of at least three independent biological replicates conducted in triplicate (*A–C*); concentration-effect relationships are the means ± SE from at least three independent biological replicates. Fold shifts above the curves denote the loss of inhibitor potency for indicated mutants *versus* WT Gq. pm, wavelength shift in picometer. DMR, dynamic mass redistribution; FR, FR900359; YM, YM-254890.

Earlier studies by us and others have introduced Gαq variants that are functional and either partially or entirely resistant to the inhibitory action of FR and YM (25, 63, 73). Yet, neither study intended to probe a causal relationship between FR action and Gq inhibition or to interrogate the occurrence of potential off-target effects, which may confound data analysis and interpretation. This distinction ideally requires a mutant designed to achieve the greatest possible loss of FR sensitivity with the minimal number of amino acid replacements. Currently available FR-insensitive Gαq mutants were generated by swapping either five or three Gαq residues for those of Gα16 and Gαs, respectively, two proteins which are naturally not FR regulated (63, 73). We reasoned that even fewer but more drastic amino acid changes may suffice to fully ablate inhibitor action, provided that they disrupt key hydrophobic forces between FR and its protein target. Guided by our own and published knowledge of the FR binding site (25, 63, 73), we predicted replacement of phenylalanine 75 (CGN: H.HA.7) and isoleucine 190 (CGN: G.S2.2) to be particularly impactful. Both residues are key components of a small cluster of nonpolar amino acids and form crucial hydrophobic interactions with FR by engaging with the N-acetyl-hydroxyleucine and the phenyllactic acid building blocks of the inhibitor (Fig. 1). We disrupted these hydrophobic forces by introducing different types of potentially unfavorable interactions using targeted mutagenesis: electrostatic repulsion at position F75 (Gαq^F75K^) and a steric clash at position I190 (Gαq^I190W^). We investigated functionality and sensitivity to FR and YM of the mutants using label-free live-cell biosensing based on dynamic mass redistribution (DMR) in HEK-ΔGq/11 cells. In this way, we restricted signaling analysis to the Gαq variant, which is introduced into cells by transient transfection, and eliminated the confounding interference caused by endogenous WT proteins (Fig. S1). Both single- and the double-mutant Gαq^F75K I190W^ preserved WT potency when activated with carbachol (CCh) *via* endogenously expressed M3 muscarinic receptors (Fig. 2, *A* and *A_i_*). Maximal signaling strength varied somewhat for the individual Gα proteins related to both variation in total cellular abundance and altered subcellular localization (Fig. S2).

FR interrupted Gq^WT^ signaling with the expected low micromolar potency and also that of the single mutants, albeit less effectively, but was almost inactive when both mutations were combined (Fig. 2, *B* and *B_i_*). For YM, potency drops were even more remarkable with full inactivity on the double mutant (Fig. 2, *C* and *C_i_*). These data agree well with earlier mutagenesis studies showing that single mutations are not sufficient to lose FR or YM inhibition, and that it is easier to perturb Gq inhibition by YM than FR (63). Thus, the exchange of two amino acids only eliminates YM inhibition of Gq and severely compromises that of FR.

### A Gαq triple mutant has no obvious comparative advantage over the double mutant

**Figure 3:**
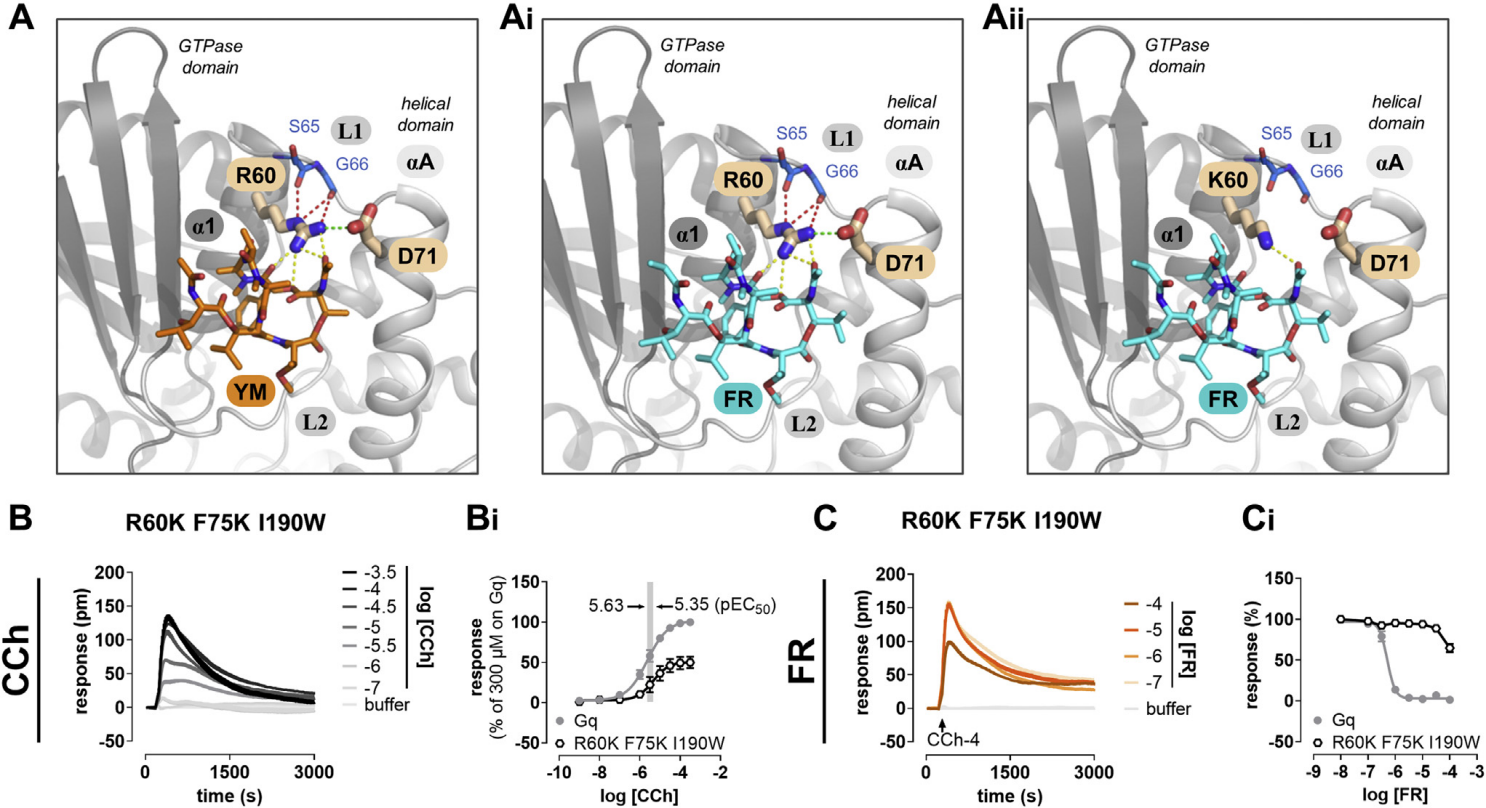
**A Gαq triple mutant has no obvious comparative advantage over the double mutant.***A–A*_*ii*_, zoomed-in view into the Gαq Asp71-Arg60 salt bridge (*green stippled line*) which stabilizes YM (*A*) and FR (*A*_*i*_) linker 1 interaction by forming multiple hydrogen bonds (*yellow stippled lines*). This hydrogen-bonding and the additional interactions with residues in linker 1 (*red stippled lines*) require the terminal guanidinium group of Arg60 and cannot take place in R60K, as evident from the fewer favorable interactions (*A*_*ii*_). All intermolecular contacts depicted as *stippled lines* are within 3.5 Å to the bound inhibitor. Oxygen and nitrogen are colored *red* and *blue*, respectively. *B*, concentration-dependent activation profiles of CCh in HEK-ΔGq/11 cells transiently transfected to express WT or mutant Gαq using label-free DMR biosensing. *B*_*i*_, concentration-effect curves of the traces depicted in (*B*), normalized to the effect of 300-μM CCh on Gq^WT^. *C*, *C*_*i*_, concentration-dependent inhibition by FR of cell responses and quantification thereof (*C*_*i*_) using 100-μM CCh. DMR recordings are representative (mean + SE) of at least three independent replicates conducted in triplicate; concentration-effect relationships are the means ± SE from at least three independent experiments. CCh, carbachol; DMR, dynamic mass redistribution; FR, FR900359; pm, wavelength shift in picometer; YM, YM-254890.

Because the double-mutant Gαq^F75K I190W^ was largely but not entirely FR resistant, we attempted to remove residual FR inhibition by targeting arginine 60 (Arg60) (CGN: G.H1.9). Arg60 is known to directly contact YM by forming multiple hydrogen bonds, to engage in a strong electrostatic interaction, a salt bridge with Asp71, and additionally to stabilize the exposed backbone of linker 1 (Fig. 3*A* and (25)). Replacement of Arg60 by lysine (R60K) is thought to disrupt this salt bridge and to severely impair Gq inhibition by YM (25). However, whether Arg-Asp salt bridge disruption and hydrogen bond disassembly would be similarly suited to ablate Gq inhibition by FR within the double mutant is unknown at present (Fig. 3, *A_i_* and *A_ii_*). To our surprise, swapping Arg60 for lysine, which had no impact on activation by CCh *via* endogenous M3 receptors (Fig. 3, *B* and *B_i_*), no further diminished the inhibitory action of FR (Fig. 3, *C* and *C_i_*). Apparently, loss of the advantageous properties of arginine over lysine, which forms fewer and less-stable ionic interactions than arginine at equivalent positions in proteins (74), and likely also at this salt bridge position (Fig. 3, *A_i_* and *A_ii_*), affects FR to a lesser extent than YM. Because the triple mutant did not offer a noticeable benefit over the double mutant concerning the function (compare Fig. 3*B_i_* with Fig. 2*A_i_*), inhibitor sensitivity (compare Fig. 3*C_i_* with Fig. 2*B_i_*), or expression (Fig. S3) and because FR inhibition occurred at exceedingly high concentrations only, which are not commonly used (24, 25, 73), we selected the double-mutant Gαq^F75K I190W^ for further mechanistic characterization.

### Canonical Gq functional and binding assays confirm resistance of the Gαq^F75K I190W^ mutant toward FR inhibition

**Figure 4:**
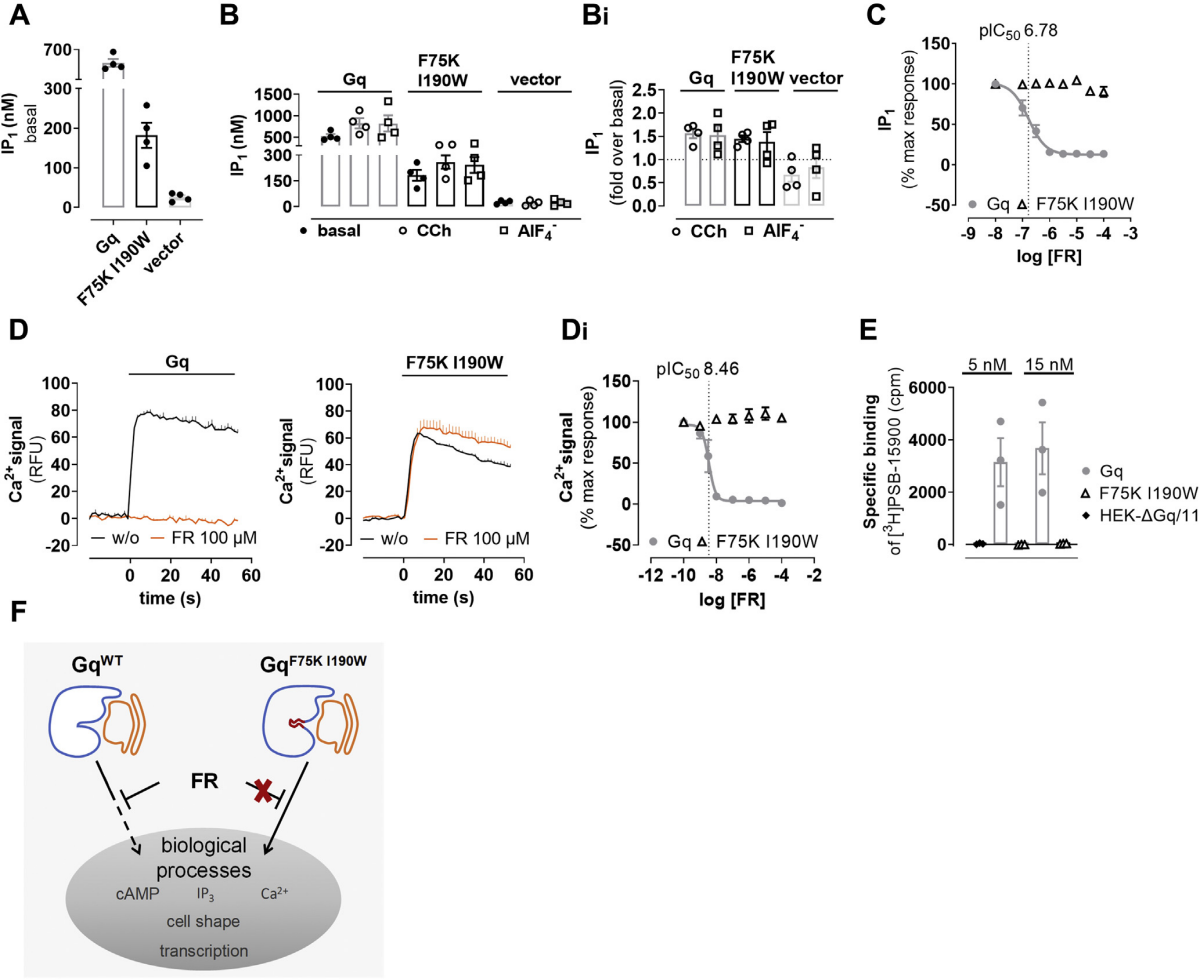
**Canonical Gq functional and binding assays confirm resistance of the Gαq**^**F75K I190W**^**mutant toward FR inhibition.***A*, intrinsic IP_1_ accumulation in HEK-ΔGq/11 cells transiently transfected with an empty vector or with plasmids encoding for Gαq^WT^ or Gαq^F75K I190W^. Data are the means ± SE of four experiments, each performed in triplicate. *B, B*_*i*_, CCh- or aluminum tetrafluoride (AlF_4_^−^)-induced accumulation of IP_1_ in HEK-ΔGq/11 cells transiently transfected with the indicated constructs. Each data point represents an independent experiment, and the bar heights show the mean ± SE of four independent experiments, each performed in triplicate. The *vertical stippled line* indicates basal IP_1_ production. *C*, FR inhibition of IP_1_ accumulation in HEK-ΔGq/11 cells transiently expressing Gαq^WT^ or Gαq^F75K I190W^. Cells were pretreated with varying concentrations of FR before stimulation with 100-μM CCh. Data are the means ± SE from at least three independent experiments each performed as technical triplicate. *D*, representative Ca^2+^ recordings in response to 100-μM CCh in HEK-ΔGq/11 cells, pretreated or not with 100-μM FR. *D*_*i*_, concentration-inhibition curves of the data shown in (*D*). Summarized data are the means ± SE of three biologically independent experiments, each performed in triplicate. *E*, specific binding in cpm of the FR-based ^3^H-labeled high-affinity tracer [³H]PSB-15900 (5 and 15 nM) to membrane preparations collected from HEK-ΔGq/11 cells transiently expressing Gαq^WT^ (15-μg protein per well) or Gαq^F75K I190W^ (50-μg protein per well). Columns represent the means ± SE of three independent experiments, each performed in triplicate. *F*, cartoon visualizing the general pharmacological principle of the drug-resistant Gαq mutant. Upon activation, both Gq^WT^ and Gq^F75K I190W^ set in motion a number of biological processes (examples in *gray*). Because FR inhibits Gq^WT^, but not Gq^F75K I190W^, it affects these processes only in cells expressing the WT isoform (*stippled line arrow*). By contrast, in cells expressing the drug-resistant variant, Gq signaling proceeds undisturbed despite the presence of FR (*solid line arrow* and *red cross* indicate the release of FR inhibition achieved with the FR-resistance mutations). As a corollary, any FR effects remaining in the presence of Gq^F75K I190W^ would be nonspecific. CCh, carbachol; FR, FR900359; IP_1_, inositol monophosphate; YM, YM-254890.

Additional approaches to interrogate Gq signaling are assays to quantify inositol monophosphate (IP_1_) accumulation or mobilization of calcium from intracellular stores (75, 76, 77, 78, 79). Of these, IP_1_ assays are particularly suited to measure both basal and ligand-activated signaling. Because F75K and I190W are located within or in close proximity to the Gα linker regions, which connect the GTPase and the helical domain (Fig. 1, *A* and *C*), and because linker flexibility is key to separation of both domains to facilitate nucleotide exchange and cellular signaling, mutations at these positions might influence intrinsic and/or receptor-mediated Gα activity. In line with the reduced cellular expression and plasma membrane abundance of Gαq^F75K I190W^ as compared with Gαq^WT^ (cf. Fig. S2), we found lower basal IP_1_ production for the double mutant (Fig. 4*A*). However, signaling capacity of Gαq^F75K I190W^ in response to a ligand-activated G_q_PCR or to aluminum tetrafluoride, which functions as pan–G protein activator, was essentially identical to that of Gαq^WT^ (Fig. 4, *B* and *B_i_*). These data indicated that the quantitative discrepancies in basal and ligand-mediated IP_1_ production are likely related to cellular expression and subcellular location differences rather than impaired G protein function or altered GPCR–G protein coupling.

As anticipated, FR inhibited signaling of Gαq^WT^ with the expected low micromolar potency in IP_1_ accumulation assays but was completely inert on the mutant, even at concentrations as high as 100 μM (Fig. 4*C*). The absence of FR inhibition at the highest applied concentration was also apparent in cytosolic calcium measurements, although these are significantly more sensitive for detecting Gq inhibition than DMR biosensing and IP_1_ accumulation assays (Fig. 4*D_i_*, compare with Figs. 4*C* and 2*B*_*i*_) (28, 64, 80). To further corroborate the apparent lack of inhibitor–protein interaction, we quantified binding of a Gq-specific FR-based ^3^H-labeled high-affinity tracer (26) to membranes prepared from HEK-ΔGq/11 cells after re-expression of Gαq^F75K I190W^ and Gαq^WT^. Entirely consistent with our functional data, we observed Gq-specific binding only in membranes collected from Gαq^WT^- but not from Gαq^F75K I190W^-expressing cells (Fig. 4*E*). Increasing the concentration of radioligand by a factor of three to accommodate for the reduced cellular expression of Gαq^F75K I190W^ did not yield any detectable specific binding for the mutant (Fig. 4*E*). On the basis of these functional and binding data, we concluded that disruption of two key hydrophobic interactions, known to be essential for tightening the ligand to its protein target, is required and sufficient to eliminate Gq inhibition by FR and YM. This finding was rather unexpected given the limited impact of single amino acid substitutions for FR and YM inhibition of Gq, which were reported in previous mutagenesis studies (63, 73). Hence, we applied Gαq^F75K I190W^ as pharmacological tool for discerning Gq-specific from non–Gq-mediated effects and thereby verify the cellular specificity of FR and YM. Consequently, Gαq^F75K I190W^ should *in vitro* be well suited to assess Gq contribution to complex cellular processes with input from multiple signaling pathways (Fig. 4*F*).

### FR and YM unmask Gq input to canonical Gs-driven cAMP production

**Figure 5:**
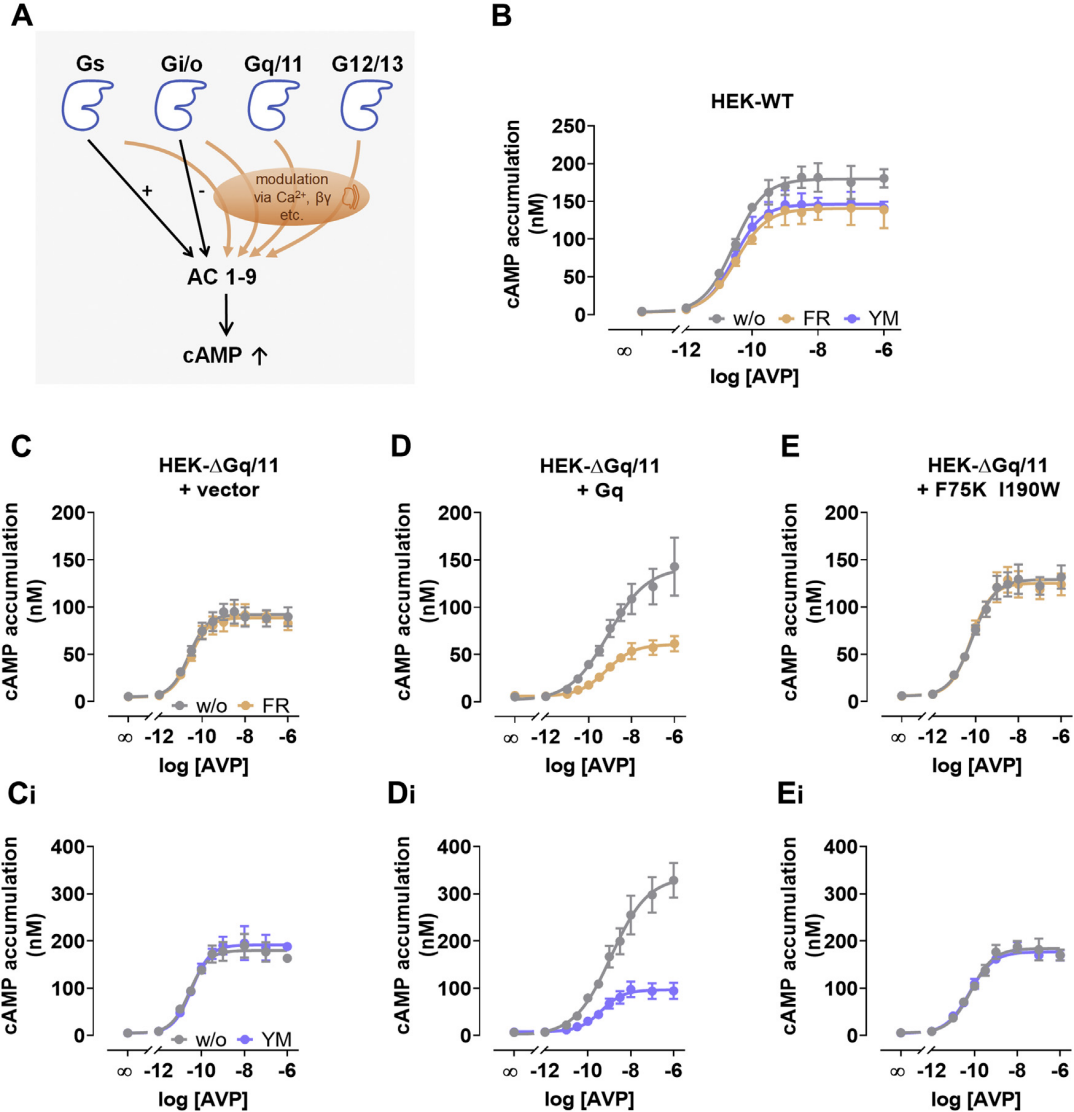
**FR and YM unmask Gq input to canonical Gs-driven cAMP production.***A*, cartoon illustrating the complexity of cAMP production. The adenylyl cyclase family of enzymes comprising the isoforms 1 to 9 (AC 1–9) catalyze formation of cAMP, and this process is influenced in various ways by G proteins of the four major Gα families (*blue*). Gαs and Gαi/o subunits exert their effects by direct stimulation or inhibition, respectively, of adenylyl cyclases (*black arrows*). Indirect modulation *via* Ca^2+^ and/or the various Gβγ subunit complexes is also possible (*orange arrows*). *B*, the effect of FR and YM (10 μM) on AVP-mediated cAMP accumulation in native HEK293 cells transiently transfected with the Gs-coupled vasopressin V2 receptor. *C–E*, the effect of FR and YM (10 μM) on AVP-mediated cAMP accumulation in HEK-ΔGq/11 cells transiently transfected with either vector control (*C*, *C*_*i*_) or the indicated Gαq constructs (Gαq^WT^ in *D*, *D*_*i*_, Gαq^F75K I190W^ in *E*, *E*_*i*_) along with the Gs-coupled vasopressin V2 receptor. Note that cAMP quantifications in the presence of YM were not generated in parallel to those with FR, accounting for the different cAMP amounts in both experimental series. Data are the means ±, +, − SE of at least three biologically independent experiments, each performed in triplicate. AVP, arginine vasopressin; FR, FR900359; YM, YM-254890.

cAMP is a second messenger, which is produced from ATP by the action of several AC isoforms to control multiple effectors such as PKA, cAMP-dependent GEFs, and cyclic nucleotide-gated channels across different time scales (81). Elevated cAMP levels are most commonly associated with activation of Gs family proteins; however, Gi/o-, G12/13-, and Gq/11-dependent signals all impair or enhance cAMP formation, often in a cell type-specific manner (Fig. 5*A*) (67, 82, 83, 84, 85, 86, 87). Because cross talk between and reciprocal reinforcement of Gs- and Gq-coupled pathways are particularly well established (88, 89, 90, 91), we anticipated FR and YM to specifically unmask Gq components, provided that they contribute to formation of cAMP by Gs- or Gq/s-coupled receptors. In line with the findings from Peng *et* *al.*, who reported YM depression of cAMP production by several G_s_PCR in human coronary artery endothelial cells (65), FR and YM diminished cAMP elevation by the Gs-sensitive V2 vasopressin receptor in HEK293 cells (Fig. 5*B*). To discern unambiguously whether inhibitor effects arose from Gs inhibition or, instead, dependence on Gq of the Gs-cAMP-AC module, we quantified V2-mediated cAMP increases in the genetic absence of Gαq and Gα11 (HEK-ΔGq/11 cells). Arginine vasopressin elicited concentration-dependent cAMP elevation in HEK-ΔGq/11 cells, and this effect was indistinguishable in the absence and presence of FR or YM, entirely congruent with the Gq-specific action of both inhibitors (Fig. 5, *C* and *C_i_*). Re-expression of Gαq^WT^ markedly amplified V2-mediated cAMP formation, indicating a possible Gq input (Fig. 5, *D* and *D_i_*). As expected, FR and YM effectively diminished the abundance of cAMP in cells expressing Gαq^WT^ (Fig. 5, *D* and *D_i_*) but were completely inert when the equivalent experiment was performed in cells expressing the inhibitor-resistant mutant Gαq^F75K I190W^ (Fig. 5, *E* and *E_i_*). These data clearly rule out direct Gs inhibition by FR and YM but, instead, corroborate their pharmacological profile in cells as Gq-specific inhibitors. FR and YM may therefore be applied to explicitly unmask Gq involvement in Gs-driven downstream signaling such as, but not limited to, production of cAMP.

### FR unmasks Gq contribution to integrated whole-cell activation profiles

**Figure 6:**
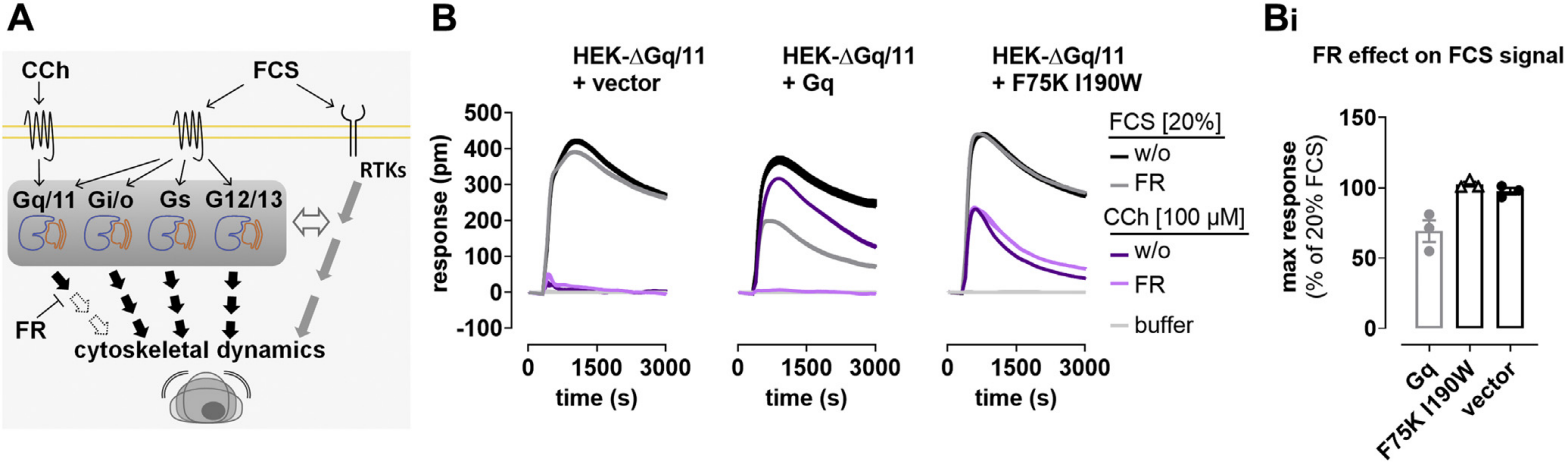
**Label-free DMR establishes specificity of FR for inhibition of Gq over non-Gq–mediated whole-cell activation.***A*, cell morphology is an integrated readout depicting the molecular underpinnings of a cell's function and thus can be used to visualize input from major signaling hubs such as G proteins and receptor tyrosine kinases (RTKs). Carbachol (CCh) exerts its effect on cytoskeletal dynamics and cell morphology by Gq signaling only, whereas fetal calf serum (FCS) is a pleiotropic stimulus, engaging multiple parallel signaling routes that involve G proteins from all four families and RTKs. Cross talk between G protein–induced and RTK-induced pathways further interconnects the two signaling branches (*double-sided horizontal arrow*). FR (*bottom left*) inhibits Gq signaling only and hence can be used to tease out the Gq contribution to cytoskeletal dynamics. *B*, DMR analysis of whole-cell responses evoked by CCh or FCS, in the presence or absence of FR (1 μM) in HEK-ΔGq/11 cells transiently expressing Gαq^WT^ or Gαq^F75K I190W^. Shown are real-time measurements (mean + SE, technical triplicates) representative of three such experiments. *B*_*i*_, quantification of the FCS signal in the presence of FR for the different constructs from the experiments shown in (*B*). DMR, dynamic mass redistribution; FR, FR900359.

Alterations of the levels of second messengers such as cAMP or Ca^2+^ often precede any detectable changes in cytoskeletal dynamics, a key determinant of the cell shape (92, 93, 94, 95, 96, 97). Activation of GPCRs from all coupling classes gives shape to cells and enables distinctly different changes of cellular morphology in a G protein subfamily–specific manner (98, 99). Label-free whole-cell biosensing based on detection of DMR is a technology platform that provides measures for precisely such morphology changes in real-time and living cells (100, 101). Here we used DMR, HEK-ΔGq/11 cells re-expressing Gαq^WT^ or Gαq^F75K I190W^, along with fetal calf serum (FCS) as the cellular response trigger. FCS was selected for its intrinsic property to initiate pleiotropic signaling *via* at least two major signaling hubs, heterotrimeric G proteins of all four subfamilies and receptor tyrosine kinases (RTKs) (102). Noncanonical activation of Gα proteins by RTKs either *via* direct protein–protein interaction or as functional cross talk in the form of transactivation is also well established (103, 104, 105, 106, 107, 108, 109, 110). Therefore, we considered FCS particularly suited to convey parallel interconnected signaling waves to tease out potential Gq involvement (Fig. 6*A*). As expected, FCS was active across all transfectants albeit with nuanced differences, while CCh promoted global cell activation in a strictly Gq-dependent manner (Fig. 6*B*). FR, on its own, did not appreciably alter cell morphology (Fig. S4), nor did it dampen the DMR response profiles of CCh and FCS in cells expressing Gαq^F75K I190W^ (Fig. 6, *B* and *B_i_*). However, in cells expressing Gαq^WT^, FR completely blunted CCh signals and partly diminished those of FCS (Fig. 6, *B* and *B_i_*). Thus, FR inhibited cellular signaling only in the presence of its biological target but was completely inert when cells were null for Gαq^WT^ or expressed the inhibitor-resistant mutant. These results again corroborate the Gq-specific action of FR and convincingly demonstrate that application of this inhibitor makes possible to tease out Gq input to complex cellular processes such as those that operate when cells change their shape in response to extracellular cues.

### FR and YM unveil Gq contribution to extracellular signal–regulated kinase/mitogen-activated protein kinase–driven transcription

**Figure 7:**
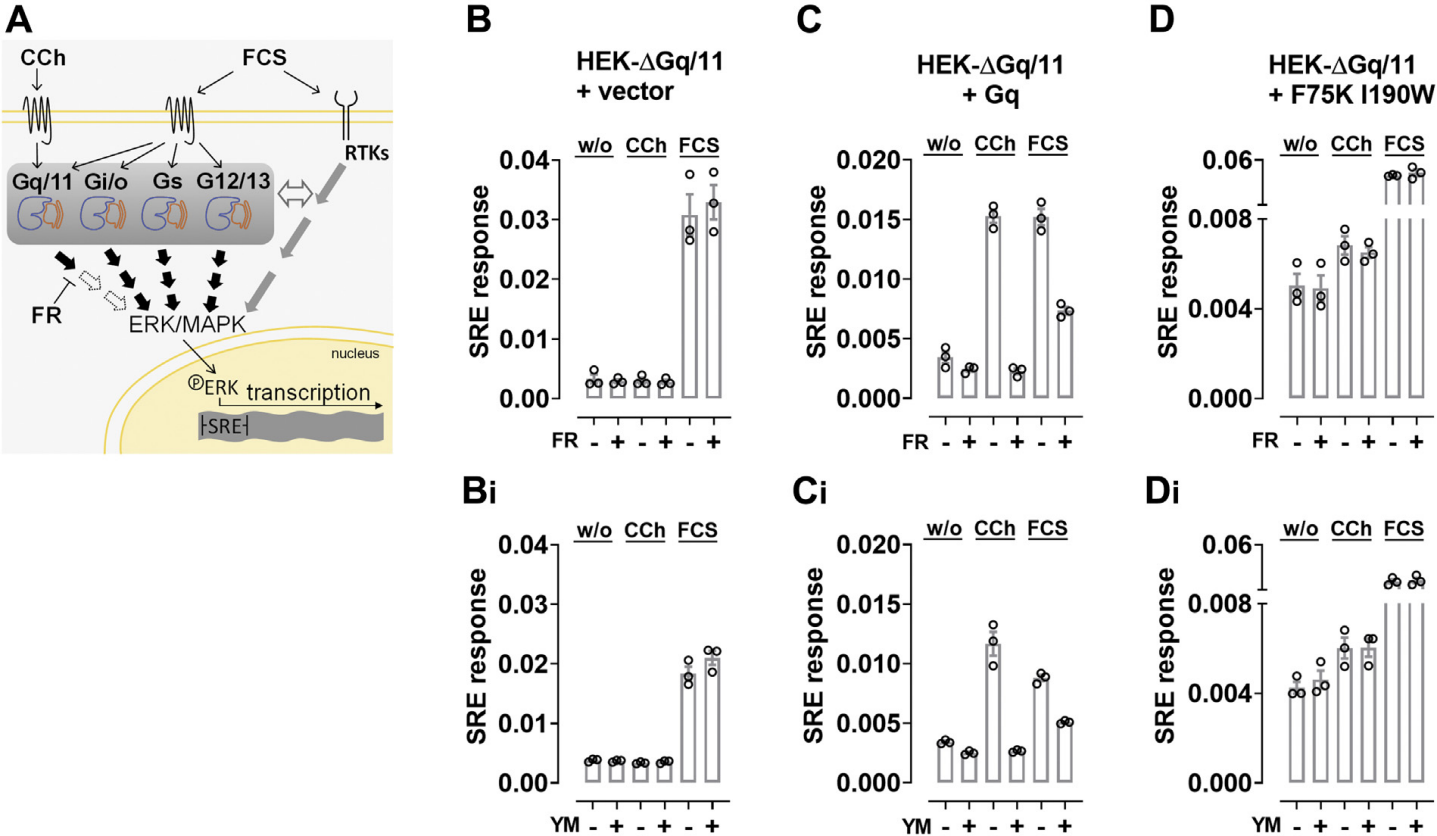
**FR and YM unveil Gq contribution to ERK/MAPK-driven transcription.***A*, the starting point of the ERK/MAPK signaling pathway is the binding of an extracellular ligand to a transmembrane protein. The resulting signaling cascade culminates with translocation of phosphorylated ERK to the nucleus to initiate transcriptional changes; here, serum response element (SRE)-driven transcription of reporter genes. CCh stimulates the cascade *via* Gq-coupled M3 receptors, whereas FCS is a pleiotropic stimulus, acting *via* multiple G proteins and RTKs. Cross talk between G proteins and RTKs further amalgamates the two signaling branches (*double-sided empty arrow*). FR (*middle left*) can be used to unveil a potential Gq component in SRE-driven transcription. *B–D*, FR and YM (1 μM) modulation of SRE-controlled reporter gene transcription in HEK-ΔGq/11 cells transfected with an empty vector (*B, B*_*i*_), Gαq^WT^ (*C*, *C*_*i*_), or Gαq^F75K I190W^ (*D*, *D*_*i*_) and stimulated with CCh (100 μM) or FCS (20%). Columns represent the means ± SE of three independent experiments, each performed in triplicate. CCh, carbachol; ERK, extracellular signal–regulated kinase; FR, FR900359; MAPK, mitogen-activated protein kinase; RTK, receptor tyrosine kinases; YM, YM-254890.

Mitogen-activated protein kinases of the extracellular signal–regulated kinase family amplify, transduce, and integrate signals from a variety of stimuli to drive transcriptional changes (111, 112, 113, 114). Reporter gene assays capture these changes and thereby offer another complex and even more downstream readout to follow signal transduction of cell surface proteins on the level of nuclear gene transcription (115). Unlike biochemical and second messenger assays, reporter gene assays are particularly susceptible to off-target responses, which explains their frequent association with prohibitively high numbers of false positives in drug-screening campaigns (116). For the very same reason, reporter gene assays should be particularly suited to pick up potential off-target effects of FR or YM. Serum response element (SRE)-driven transcription dependent on extracellular signal–regulated kinase nuclear translocation is one such approach that collates the signaling of all major G protein families, and of RTKs, within a single transcriptional readout (102, 117, 118, 119, 120, 121). Here, we took advantage of CCh to produce single-pathway input and of FCS to produce multipathway input in SRE assays (Fig. 7*A*). As expected, stimulation of HEK-ΔGq/11 cells with FCS, but not with CCh, induced robust elevation of SRE activity, and this effect was insensitive to FR or YM pretreatment (Fig. 7, *B* and *B_i_*). Re-expression of Gαq^WT^ in the KO background enabled both CCh- and FCS-mediated transcription of SRE-regulated reporter activity (Fig. 7, *C* and *C_i_*). FR and YM completely blunted the CCh-induced transcriptional changes and markedly reduced those of FCS, in line with convergent signaling by FCS through Gq and non-Gq pathways (Fig. 7, *C* and *C_i_*). Conversely, FR and YM were ineffective in cells expressing Gαq^F75K I190W^ regardless of whether CCh or FCS were applied as activating stimuli (Fig. 7, *D* and *D_i_*). These data again attested the inhibitors' high Gq selectivity and clearly categorized their effects as ‘on-target.’ We concluded FR and YM are no broad-spectrum G protein inhibitors but highly selective for G protein heterotrimers containing Gαq family subunits.

### FR underlines Gq requirement for Gi-Gβγ calcium but is no general Gβγ signaling inhibitor

**Figure 8:**
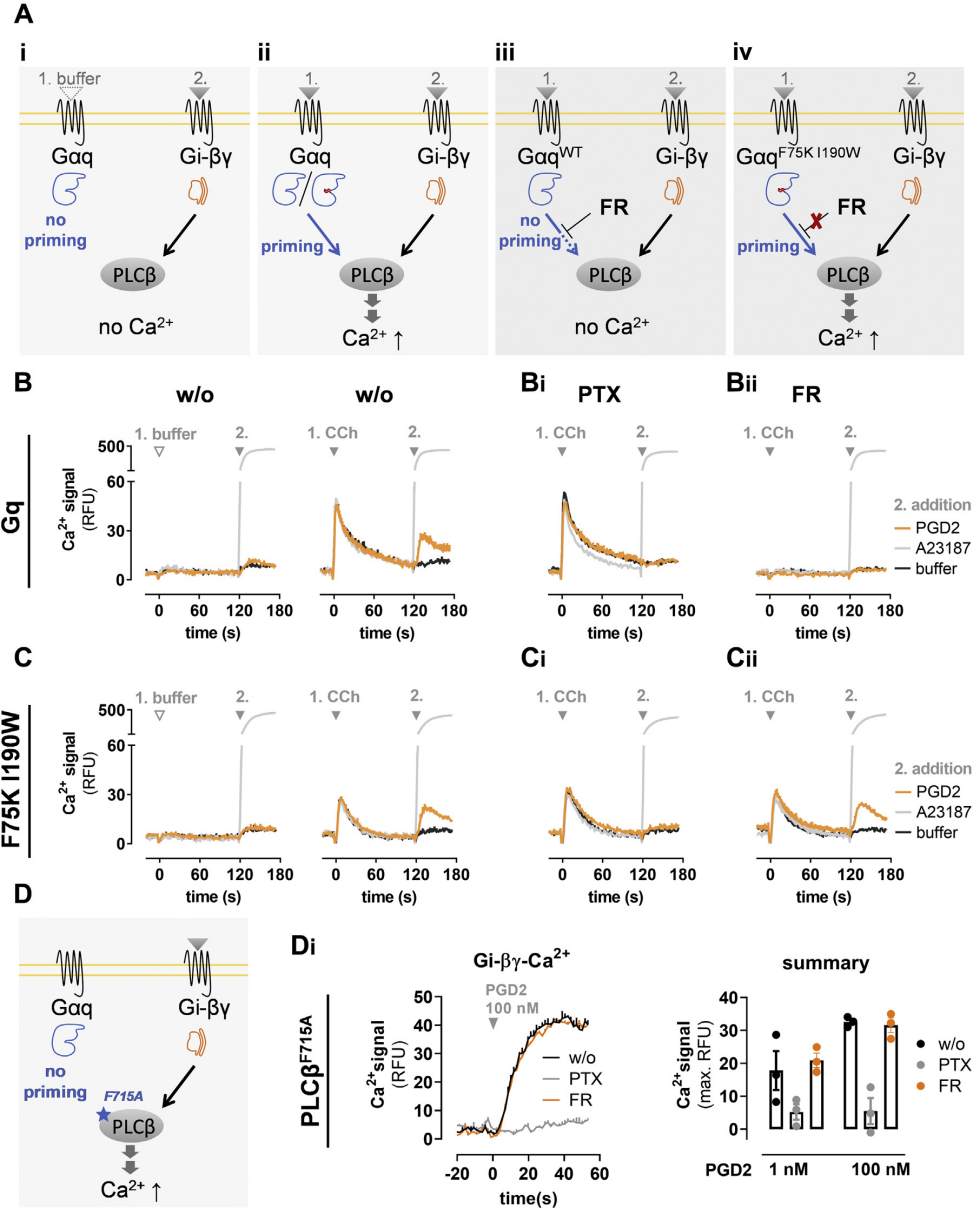
**FR underlines Gq requirement for Gi-Gβγ calcium but is no general Gβγ signaling inhibitor.***A*, priming of phospholipase Cβ (PLCβ) by active Gαq (*blue*) is necessary for its activation by Gi-derived βγ (*orange*). *A*_*i*_, without priming, binding of Gi-βγ to PLCβ does not lead to a release of calcium ions from intracellular stores. *A*_*ii*_, both active Gαq^WT^ and Gαq^F75K I190W^ can stimulate and prime PLCβ. This priming enables Gi-βγ to activate PLCβ followed by an increase in Ca^2+^. *A*_*iii*_, FR inhibits Gq and, in doing so, interdicts priming of PLCβ by active Gαq. Consequently, Gi-βγ does not mobilize Ca^2+^*via* PLCβ enzymes. *A*_*iv*_, FR does not hinder signaling of the inhibitor-resistant Gαq^F75K I190W^ variant, and therefore, priming of PLCβ, the mandatory requirement to enable Gi-βγ-Ca^2+^, occurs undisturbed despite the presence of FR. *B* and *C*, HEK-ΔGq/11 cells transiently expressing the D prostanoid receptor 2 receptor together with Gαq^WT^ (*B*) or Gαq^F75K I190W^ (*C*) were pretreated as indicated and Ca^2+^ transients were monitored following a two-step sequential compound addition protocol. At t = 0, either buffer or Gq stimulus CCh (10 μM) was added to all cells. At t = 120 s, the indicated compound or buffer was applied. *D*, priming of the phospholipase Cβ^F715A^ mutant (PLCβ) by active Gαq (*blue*) is not necessary for its activation by Gi-derived βγ (*orange*). *D*_*i*_, HEK cells expressing a variant of the D prostanoid receptor 2 receptor carrying a C-terminal deletion (136) along with PLCβ^F715A^ were pretreated as indicated. Then, calcium mobilization was measured upon stimulation with PGD2. The *left panel* shows representative calcium kinetics quantified in the *right panel*. All traces are the mean +SE representative of three such experiments, each performed in duplicate. Columns represent the means ± SE of three independent experiments, each performed in duplicate. A23187 (aka calcium ionophore) (5 μM) was used as a viability control in all experiments. CCh, carbachol; FR, FR900359.

Gi-Gβγ calcium is a signaling paradigm coined to reflect calcium mobilization by ligand-activated Gi/o-coupled GPCRs. Therein, G_i/o_PCRs rely on Gi-derived Gβγ to activate PLCβ isoforms that convert plasma membrane phosphatidylinositol 4,5-bisphosphate into the second messengers DAG and IP3, the latter mobilizing Ca^2+^ from endoplasmic reticulum stores (122, 123). A hallmark feature of this Gi-βγ-PLCβ-Ca^2+^ signaling module is its sensitivity to pretreatment with Gi/o inhibitor PTX (124, 125). We and others (64, 124) recently reported that Gi-Ca^2+^ is also fully blocked when cells are pretreated with FR or YM. FR sensitivity of Gi-βγ-Ca^2+^ but not of Gi-mediated cAMP lowering has led some authors to hypothesize that FR and YM might inhibit Gβγ signaling as well (64, 65). However, this finding may be subject to alternative interpretation (FR inhibits Gβγ *versus* Gq is required for Gi-Gβγ-calcium), which is why we previously investigated the molecular mechanism whereby this inhibition occurred (124). We found that FR is no general Gβγ inhibitor because it does not dampen Gβγ-activated G protein–coupled inwardly rectifying potassium channels (124). Rather, Gi-calcium is fully dependent on Gq activation in the living cell context, which explains why Gi-calcium is undetectable without Gq priming (Fig. 8*A_i_*), measurable when a Gq stimulus is applied before or concomitant with a Gi stimulus (124) (Fig. 8*A_ii_*), and absent in cells pretreated with FR (Fig. 8*A_iii_*). However, in cells expressing Gαq^F75K I190W^, Gi-calcium should be retained despite the presence of FR (Fig. 8*A_iv_*). Conversely, extinction of Gi-calcium also in Gαq^F75K I190W^-expressing cells would indicate targeting by FR of Gi-derived Gβγ. In line with our previous findings, Gi-calcium evoked with prostaglandin D2 *via* Gi-coupled D prostanoid receptor 2 occurred only after priming with CCh to provide Gq input (Fig. 8*B*). Pretreatment with Gi inhibitor PTX specifically disabled Gi-calcium (Fig. 8*B_i_*), while FR pretreatment disabled the permissive Gq peak and, consequently, prevented occurrence of the second Gi peak (Fig. 8*B_ii_*). The permissive action of CCh for Gi-calcium was similarly visible in Gαq^F75K I190W^-expressing cells (Fig. 8*C*), where PTX consistently erased Gi-calcium (Fig. 8*C_i_*) while FR failed to abolish both, the first Gq peak and the second Gi peak (Fig. 8*C_ii_*).

PLCβ enzymes are effectively autoinhibited, and a number of proteins including Gαq release this autoinhibited state (126, 127). PLCβ3–F715A is a mutant variant that no longer demands Gq for hydrolysis of its membrane substrate and, consequently, triggers Gi-Gβγ–calcium without the need of Gq priming (Fig. 8*D*, (124, 127)). As anticipated, HEK293 cells transfected to express PLCβ3–F715A along with D prostanoid receptor 2 responded with robust Ca^2+^ traces that were wholly blocked by PTX but unaffected by FR (Fig. 8*D_i_*). These data provided compelling evidence that Gi-derived Gβγ is no target for FR. Instead, FR abolishes Gi-Gβγ–calcium only by specific inhibition of Gq.

More generally, all our above data clearly illustrate the experimental power of the pair of a WT and engineered Gαq variant to distinguish, unambiguously, causal from observational FR and YM effects. Thereby, we do not only provide a versatile experimental strategy for probing on-target Gq *versus* off-target non-Gq action of these valuable natural product inhibitors but also a versatile experimental *in vitro* platform to decode Gq contribution to signal transduction in living cells.

## Discussion

An important prerequisite for utilizing bioactive molecules effectively as probes for biological processes is that they confidently engage their designated molecular targets only. Suboptimal target selectivity of chemical probes hampers data analysis and introduces confounding variables to data interpretation. FR and YM, two naturally occurring depsipeptides, are used widely with the implicit assumption that their cellular action is mediated *via* inhibition of Gq (24, 25, 27). Although there is scientific consensus on the Gq inhibition, the absence of potential off-target effects for FR and YM remains to be fully demonstrated. This is all the more important because recent studies surmised FR to also inhibit non-Gq events such as cAMP elevation induced by Gs-coupled GPCRs (65) or Gβγ signaling originating from GPCR-activated Gαi/o-Gβγ heterotrimers (64). Conjectures like these indeed question which of the reported effects of FR are attributable to Gq inhibition and which arise from nonspecific interaction with other Gα proteins or, potentially, completely distinct cellular targets. The central research question therefore is whether FR and YM are indeed not as selective as previously anticipated (24, 25, 27) or, whether inhibition by FR or YM of biological responses may, instead, indicate necessity of and/or specific contribution by Gq.

The approach presented in this study attempts to fill this knowledge gap by providing a molecular toolbox for unambiguous discrimination between on-target Gq and off-target non-Gq action for the two widely applied natural product inhibitors (14, 29, 30, 31, 32, 33, 34, 35, 36, 37, 38, 39, 40, 41, 42, 43, 44, 45, 46, 47, 48, 49, 50, 51, 52, 53, 54, 55, 128). One of its core elements is Gαq^F75K I190W^, a drug-resistant Gαq mutant, which is catalytically active, yet entirely refractory toward inhibitor action. If combined with Gαq^WT^, FR or YM, two ‘chemicals’ that can be added at will to act quickly, as well as cells that are null for Gαq, Gα11 and Gα14, it may be exploited to uncover whether inhibitor action occurs with the requisite specificity, that is, *via* its cognate cellular target Gq.

Here we have exploited this molecular toolbox to probe FR selectivity and Gq involvement in a number of highly complex cellular processes, characterized by multiple interdependent signaling inputs that converge in a joint signaling output: cAMP accumulation, mobilization of calcium from intracellular stores, cell morphology changes, and nuclear gene transcription. In all instances did we obtain a highly consistent picture: FR and YM produced biological effects only in the presence of Gαq. In no instance did we observe any overt signs for non-Gq action, even if inhibitors were applied at exceedingly high concentrations (that are not commonly used (24, 25, 73)), or in reporter gene assays, which are particularly prone to off-target liabilities because of their requirement for extended incubation times (115, 116). From these findings, we inferred that FR and YM are remarkably selective for Gq and that their propensity to introduce confounding variables to interpretation of biological data may be minimal at best.

Inhibitor-resistant Gαq variants have been developed in prior studies (25, 40, 63, 73), yet they have not been used to form testable hypotheses as to whether inhibitors act on-target with the requisite specificity. Our study was initiated with the core assumption that FR and YM are Gq selective, a notion that is supported by multiple lines of independent evidence. For instance, the co-crystallization of Gαq in complex with YM (25) as well as several recent mutagenesis studies (25, 63, 73) pinpointed a number of residues that are crucial for inhibitor-target interaction, but most of these residues are absent in non-Gq family Gα subunits (25). Moreover, swapping of key amino acids within the FR binding domain to their Gαs, Gαi, and Gα16 counterpart sequences rendered the mutant Gαq variants insensitive to FR or YM inhibition (40, 63, 73). By analogy, the most obvious implication of these findings is that the WT versions of Gαs, Gαi, and Gα16 are, per se, inert to FR. Beyond that, Gαs, Gαi, and Gα16 variants with artificial FR sensitivity have been created previously by introducing an engineered FR binding site (40, 63, 73). Another strong argument against nonspecific binding of FR and YM to unidentified sites on non-Gq proteins is the lack of detectable radioligand binding of FR- and YM-based radiotracers in membrane preparations from HEK-ΔGq/11 cells (26).

So, how come that FR and YM action are taken to be nonselective? The most straightforward explanation is that alternative interpretations rather than dissimilar experimental data account for this apparent discrepancy. Our study provides researchers with an experimental toolbox for careful design of well-controlled and systematic *in vitro* investigations into Gq contribution to complex biological processes. Our study also provides the experimental data to resolve this apparent discrepancy and to suggest the following consensus view: FR and YM act in cells by specific inhibition of their canonical targets Gq, G11, and G14. In other words, their biological action in cells indicates necessity of, or input from, Gq in signaling networks that cooperate with other G protein classes. Therefore, we envisage that the here-described G protein–ligand system will hold great promise to be applied to additional cell types to provide insight into the complex interplay between Gq and the remaining G protein pool in cellular regulation.

One caveat deserves further mention here: the entire study was carried out in HEK293 cells, which are among the most commonly used cell lines in biology and biotechnology. Yet, because off-target effects are context dependent and unpredictable, it is conceivable that the absence of non-Gq effects of FR in this cell line was only fortuitous. Notwithstanding this caveat, inhibitor-resistant Gαq^F75K I190W^ provides a path forward to unambiguously address such liabilities and to expedite studies on FR and YM selectivity in potentially any cellular context that is amenable to lipid- or virus-mediated transfection.

A second, probably more minor caveat for *in vitro* studies is that the Gαq^F75K I190W^ construct shows altered subcellular localization. Yet, in no instance did the cellular distribution pattern of the FR- and YM-resistant mutant preclude unambiguous assessment of inhibitor specificity or off-target effects. However, if *in vivo* studies are envisioned, involving the generation of mutant mouse lines that express the transgene at a physiological level using the WT locus, identification of inhibitor-resistant proteins with both WT catalytic activity and cellular distribution in the relevant cellular background will become an important asset.

## Conclusions

Heterotrimeric G proteins are the core transducers for ligand-activated GPCRs relaying extracellular signals to a plethora of intracellular effectors. Their involvement in numerous biological processes explains both the need and the challenge to identify, unambiguously, which G protein family is responsible for a given physiological effect. One strategy to address this challenge is by development and application of cell-permeant inhibitors that specifically interact with defined G protein subfamilies and to examine their effects in intact cells, tissues, organs, and even *in vivo*. Inhibitor experiments may deliver strong clues as to protein function, provided that they bind to their intended protein targets only, and hence, this strategy crucially depends on inhibitor selectivity. Lack of selectivity is inherent to numerous purportedly selective pharmacological tools that are being used in signal transduction research, implying that their cellular modes of action are often by far more complicated than usually assumed (129, 130, 131, 132, 133). FR and YM, on the contrary, stand out unique for their remarkable selectivity to shut off the signaling of Gq family proteins only. Therefore, it is tempting to speculate that the here-proposed strategy may even be applicable for *in* *vivo* investigations as opposed to, or, in addition to transformed or transfected cell lines as used herein.

## Experimental procedures

### Reagents

Cell culture materials were purchased from Invitrogen, FR900359 (previous commercial name UBO-QIC) was isolated and purified as described elsewhere (24), YM-254890 was from Wako Chemicals GmbH. Primary antibodies to detect the human influenza hemagglutinin (HA) epitope tag (YPYDVPDYA) and α-tubulin were from Roche Applied Science and LifeSpan BioSciences, respectively. The horseradish peroxidase–conjugated secondary antibodies, goat anti-mouse IgG, and goat anti-rabbit IgG were from Sigma-Aldrich and Antibodies-online GmbH, respectively. All other reagents were purchased from Sigma-Aldrich if not stated otherwise.

### Cell culture

All cell lines were cultivated in Dulbecco's modified Eagle's medium with 5% CO_2_ at 37 °C in a humidified atmosphere. The medium was supplemented with 10% (v/v) FCS, penicillin (100 U/ml), and streptomycin (0.1 mg/ml). Generation of genetically engineered HEK293 cells using CRISPR-Cas9 technology to knock out the subunits of Gαq and Gα11 (HEK-ΔGq/11 cells) is described elsewhere (24). Cell lines were screened by PCR on a monthly basis for *mycoplasma* contamination and were tested negative.

### Site-directed mutagenesis and transfection

Mutations of the HA-tagged mouse Gαq cDNA, in pcDNA3.1(+), were generated by QuikChange site-directed PCR mutagenesis with specific primers as detailed in Table S1. Successful mutations were verified by DNA sequencing. Subconfluent cell cultures were transiently transfected with the respective expression plasmids using the polyethylenimine reagent (Polysciences) following the protocol provided by the manufacturer.

### Label-free DMR assays

DMR measurements were performed as described previously (98, 99, 134) using the Epic biosensor (Corning) together with the CyBi-SELMA semiautomated electronic pipetting system (Analytik Jena AG). In brief, 24 h after transfection with the indicated G protein α subunits, HEK-ΔGq/11 cells were counted and seeded at a density of 17,000 cells per well on 384-well fibronectin-coated biosensor plates. On the next day, cells were washed twice with Hanks' buffered salt solution (HBSS) containing 20-mM Hepes (HBSS + Hepes) and incubated for 1 h at 37 °C in the EPIC reader. FR or YM was added 1 h before the measurement in HBSS + Hepes. The sensor plate was scanned to record a baseline optical signature until cells were equilibrated, followed by agonist addition and monitoring of DMR alterations for at least 3000 s at 37 °C.

### IP_1_ accumulation assay

IP_1_ accumulation was quantified using Cisbio's homogeneous time resolved fluorescence (HTRF) technology according to the manufacturer's instructions. Cells were washed in PBS and resuspended in an assay buffer containing LiCl to prevent IP_1_ degradation. Cell numbers were adjusted to 25,000 cells per well to yield IP_1_ amounts in the linear range of the assay kit. FR was preincubated with cells for 1 h followed by 30 min of CCh or aluminum tetrafluoride stimulation. HTRF ratios of each individual well were read on the HTRF-compatible Mithras LB 940 multimode plate reader (Berthold Technologies) and were used to calculate IP_1_ abundance in nM.

### cAMP accumulation assay

For cAMP assays, the Cisbio HTRF kit (Cisbio) was used according to the manufacturer's instructions, with the following modifications: FR or YM was preincubated with cells (5000 cells/well) for 1 h followed by 30 min of arginine vasopressin stimulation. Then, the lysis buffer and HTRF components were added and incubated at room temperature for at least 1 h. HTRF ratios were read in each individual well with the Mithras LB 940 multimode plate reader (Berthold Technologies) and were converted to cAMP concentrations in nM based on a standard curve generated from the cAMP standard solutions of the manufacturer.

### Cell population–based Ca^2+^ mobilization measurements

Release of calcium from intracellular stores was measured using the FLIPR Calcium 5 assay kit (Molecular Devices) according to manufacturer's instructions. Briefly, HEK-ΔGq/11 cells were seeded in flat-bottom 96-well cell culture plates (60,000 cells per well) and cultivated overnight. The next day, the media was removed and cells were incubated with 50 μl/well Calcium 5 dye at 37 °C for 45 min (FR was dissolved in Calcium 5 dye). Afterward, the dye was diluted with 150-μl HBSS supplemented with 20-mM Hepes (100 μl for experiments using two additions). Ca^2+^ mobilization was measured as increase in fluorescence over time, using the FlexStation 3 MultiMode Benchtop reader (Molecular Devices). After an initial baseline read of 20 s, 50-μl compound was added either once after 20 s or twice at 20 s and 140 s, respectively. Five micromolar A23187 was used as a viability control in all experiments. For all assays, the first compound addition was set to *x* = 0, *y* = 0. The results show increase in [Ca^2+^]_i_ as relative fluorescence units over time.

### Western blotting

HEK-ΔGq/11 cells were seeded in cell culture plates 24 h before transfection with the plasmids of interest. Forty-eight hours after transfection, cells were washed twice with PBS and lysed in ice-cold lysis buffer (25-mM Tris, pH 7.4, 150-mM NaCl, 1-mM EDTA, 1% Triton X-100, 1% IGEPAL) supplemented with protease inhibitor mixture (Sigma). Lysates were rotated for 20 min at 4 °C and centrifuged at 15,000*g* (4 °C, 10 min). The protein concentration was determined with the Pierce BCA Protein Assay (Thermo Fisher Scientific) according to manufacturer's instructions. Lysates (20 μg of protein) were separated by 10% SDS-PAGE and transferred to a nitrocellulose membrane (Hybond-C Extra, GE Healthcare) by electroblotting. After washing with PBS containing 0.1% Tween, membranes were blocked with ROTI-Block (1×; Carl Roth) for 1 h at room temperature and incubated overnight at 4 °C in ROTI-Block with antibodies specific for the HA tag (1:1000). Membranes were washed three times with PBS containing 0.1% Tween and then incubated for 1 h at room temperature with an HRP-conjugated secondary goat anti-mouse IgG antibody (1:5000) in Roti-Block. For signal development of the immunoreactive proteins, the Amersham Biosciences ECL Prime Western blotting detection reagent (GE Healthcare) was used. To normalize for equal loading and protein transfer, membranes were reprobed with an antibody against α-tubulin (1:2000) and visualized after incubation with an HRP-conjugated secondary goat anti-rabbit IgG antibody (1:10,000). Quantification of the immunoreactive bands was carried out by densitometry using ImageJ (135) (National Institutes of Health).

### Structural analysis and homology models

All structural analyses are based on the Gq–YM crystal structure (Protein Data Bank [PDB] code: 3AH8). The propionyl and isopropyl substituents of FR were manually added to the inhibitor in PyMOL 2.0.6 (Schrödinger). Amino acid exchanges F75K, R60K, and I190W were introduced using the mutagenesis wizard in PyMOL.

### Radioligand binding assay

Binding assays were performed in membranes collected from HEK-ΔGq/11 cells after transient transfection with Gαq^WT^ or Gαq^F75K I190W^ using the FR-based radiotracer [³H]PSB-15900 (50-mM Tris HCl, pH 7.4; 200-μl final assay volume). Each assay tube contained 50-μl [³H]PSB-15900 at a final concentration of 5 and 15 nM, respectively, 50 μl of membranes in the assay buffer (corresponding to either 15 μg of Gαq^WT^ or 50 μg of Gαq^F75K I190W^ to compensate for its lower cellular abundance), 95-μl assay buffer, and 5 μl of dimethyl sulfoxide. Nonspecific binding was determined in the presence of 5-μM FR. The samples were incubated at 37 °C for 90 min with gentle shaking, and the reaction was started by the adding the membrane preparations. Incubation was terminated by rapid filtration through GF/C glass fiber filters using a Brandel 24-well harvester. Filters were dried, punched out, transferred to scintillation vials, and incubated for at least 6 h in scintillation cocktail (2.5-ml ProSafe FC+, Meridian Biotechnologies Ltd) before liquid scintillation counting (Thermo Fisher Tri-Carb 2810TR) at an efficiency of 55%. The radiolabeled FR-derivative [³H]PSB-15900 and HEK293 cell membranes were prepared as described previously (26).

### SRE assays

HEK-ΔGq/11 cells were transiently transfected with expression plasmids encoding Gα proteins, luciferase reporter vectors pGL4.33[luc2P/SRE/Hygro], and pCMV-Renilla (DNA ratios, 5:3:0.3). 24 h later, cells were seeded at a density of 50,000 cells per well in 96-well fibronectin-coated white opaque plates. The following day, cells were starved for at least 1 h and incubated with FR, YM, or vehicle for 60 min before treatment with an agonist or vehicle. Six hours later, the cells were exposed sequentially (10 min each) to (i) Dual-Glo Luciferase reagent (Promega), followed by quantitation of the firefly luciferase reaction (light A), and (ii) Stop & Glo reagent (Promega) to quench firefly luciferase luminescence but activate Renilla luciferase (light B). Luminescence measurements were taken with a Mithras LB 940 multimode reader (Berthold Technologies).

### Imaging

Microscopy was carried out on an Axio Observer inverted fluorescence microscope (Zeiss). For imaging of HA-tagged Gαq proteins, transiently transfected HEK-ΔGq/11 cells were seeded onto PDL-coated 8-well μ-slides with a glass bottom (ibidi). The next day, cells were fixed with 4% paraformaldehyde for 20 min at room temperature, permeabilized, and blocked in blotto (PBS with 0.3% Triton X-100, 10% goat serum, and 1% bovine serum albumin) before immunostaining with an antibody recognizing the HA tag (1:500) for 1 h at 37  °C. After washing three times with PBS for 10 min at 37 °C, cells were stained with a FITC-conjugated anti-mouse antibody (1:500) for 1 h at 37  °C. Cells were washed three times with PBS for 10 min at 37 °C, and HA-tagged Gαq proteins were visualized by fluorescence microscopy using a Plan-Apochromat x63/1.40 Oil DIC and the filter set 38 (green).

### Data analysis

All data were analyzed using GraphPad Prism 8.0.0 software (GraphPad Inc). Quantification of DMR signals was performed by calculation of the maximum responses. Data points from concentration–response or concentration–inhibition curves were fitted to the following four-parameter logistic function:

$$Y=bottom\;+\;\frac{\left(top-bottom\right)}{1\;+\;10^{\left(logEC_{50}-x\right)slope}}$$

Concentration–response curves were normalized by setting each experimental maximal effect to 100%. All data are shown as the mean + or ± SEM of at least three independent experiments performed in technical duplicates or triplicates as specified in the legends.

### Data availability

All data underpinning this publication are contained within the main text and the supporting information. All raw data are available upon reasonable request from the corresponding author (kostenis@uni-bonn.de).

## Supporting information

This article contains supporting information.

## Conflict of interest

The authors declare that they have no conflict of interest with the contents of this article.
