# Supplementary material for: An experimental strategy to probe Gq contribution to signal transduction in living cells
Source: J Biol Chem. 2021 Feb 25;296:100472. doi: 10.1016/j.jbc.2021.100472 (PMC8024710; doi:10.1016/j.jbc.2021.100472)
Supplement: Figures S1 to S4 and Table S1 [file mmc1.pdf]

SUPPORTING INFORMATION

An experimental strategy to probe Gq contribution to signal transduction in living cells

Julian Patt[1], Judith Alenfelder[1], Eva Marie Pfeil[1], Jan Hendrik Voss[2], Nicole Merten[1], Funda Eryilmaz[1], Nina Heycke[1], Uli Rick[1], Asuka Inoue[3], Stefan Kehraus[4], Xavier Deupi[5], Christa E. Müller[2], Gabriele M. König[4], Max Crüsemann[4] & Evi Kostenis[1*]

[1]Molecular, Cellular and Pharmacobiology Section, Institute for Pharmaceutical Biology, University of Bonn, Nussallee 6, 53115 Bonn, Germany.

[2]PharmaCenter Bonn, Pharmaceutical Institute, Pharmaceutical & Medicinal Chemistry, University of Bonn, An der Immenburg 4, 53121 Bonn, Germany.

[3]Graduate School of Pharmaceutical Sciences, Tohoku University, 6-3, Aoba, Aramaki, Aoba-ku, Sendai, Miyagi 980-8578, Japan.

[4]Institute for Pharmaceutical Biology, University of Bonn, Nussallee 6, 53115 Bonn, Germany.

[5]Laboratory of Biomolecular Research and Condensed Matter Theory Group, Paul Scherrer Institute, 5232 Villigen, Switzerland.

*To whom correspondence should be addressed: Prof. Evi Kostenis; Molecular, Cellular and Pharmacobiology Section, Institute for Pharmaceutical Biology, University of Bonn, Nussallee 6, 53115 Bonn, Germany. kostenis@uni-bonn.de.

Running title: *Probing the cellular specificity of FR900359 and YM-254890*

The Supporting information contains:

Figure S1	**HEK-ΔGq/11cells respond to carbachol (CCh) only after re-expression of Gαq.**	page 3
Figure S2	**Decreased cellular expression and altered subcellular localization explain the reduced signaling strength of F75K harboring Gαq variants.**	page 4
Figure S3	**The FR-resistant triple and double mutant show similar cellular expression.**	page 6
Figure S4	**FR does not alter cell morphology in the absence of its biological target Gq.**	page 7
Table S1	**Primers for site-directed mutagenesis.**	page 8

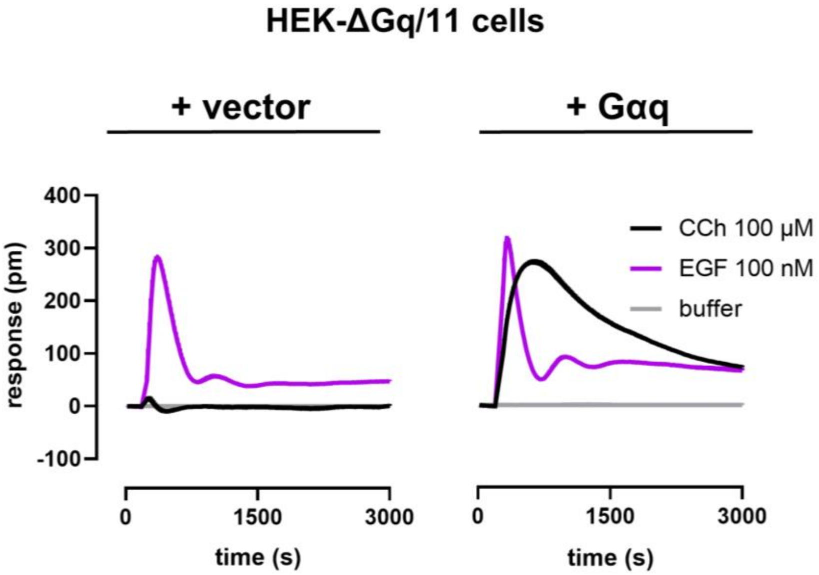

Figure S1. HEK-ΔGq/11 cells respond to carbachol (CCh) only after re-expression of Gαq. DMR analysis of whole cell responses evoked by epidermal growth factor (EGF, as the cell viability control) and carbachol (CCh), which activates Gαq-sensitive endogenous muscarinic M3 receptors, at the indicated concentrations in HEK293 cells genome-edited by CRISPR-Cas9 to lack all functional alleles for Gαq and Gα11. HEK-ΔGq/11 cells respond to epidermal growth factor (EGF) with robust alteration of DMR profiles but require the presence of Gαq to respond to CCh. Shown are real-time measurements (mean + S.E., technical triplicates) representative of three such experiments.

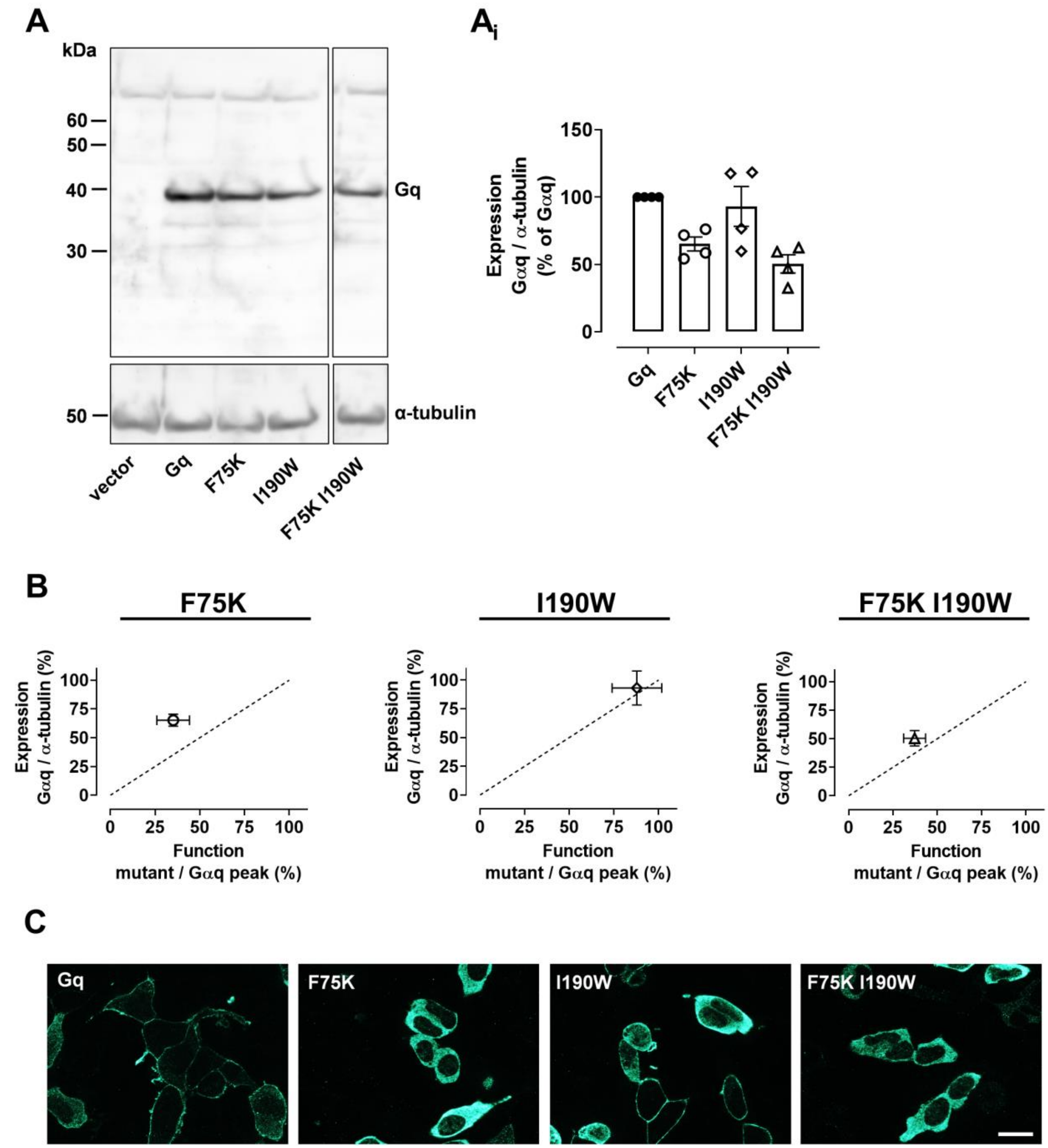

Figure S2. Decreased cellular expression and altered subcellular localization explain the reduced signaling strength of F75K harboring Gαq variants. A. Representative western blot showing expression levels of Gαq^{WT} and mutant proteins detected in cellular lysates that were collected from CRISPR-Cas9 HEK-ΔGq/11 cells transiently expressing the indicated constructs and **(A_i)** quantification thereof. α-tubulin was used as the loading control. Three additional blots from independent transfections gave similar results. Images were spliced together to re-order lanes and to remove irrelevant samples. The right outermost lane on this blot is shown again in Figure S3 as lane 3. Columns represent means ± S.E. of

four independent experiments. **B.** Correlation of α-subunit function (normalized maximal DMR amplitude in response to a saturating CCh concentration of 300 µM) with total cellular abundance is shown for each mutant relative to $G\alpha q^{WT}$ control. The stippled line indicates a positive linear relationship between function and expression with margins set by vector control and $G\alpha q^{WT}$ which are arbitrarily denoted as 0 and 100, respectively. Reduced cellular expression does not fully explain the diminished signaling strength of $G\alpha q^{F75K}$ and $G\alpha q^{F75K\ I190W}$. Data are means ± S.E. of 4 experiments. **C.** Representative fluorescence microscopy images collected in CRISPR-Cas9 HEK-ΔGq/11 cells showing cellular localization of the individual Gα constructs. Overexpressed $G\alpha q^{WT}$ and $G\alpha q^{I190W}$ are largely restricted to the plasma membrane, while $G\alpha q^{F75K}$ and $G\alpha q^{F75K\ I190W}$ were predominantly intracellular. One of three independent experiments is shown. Scale bar = 20 µm.

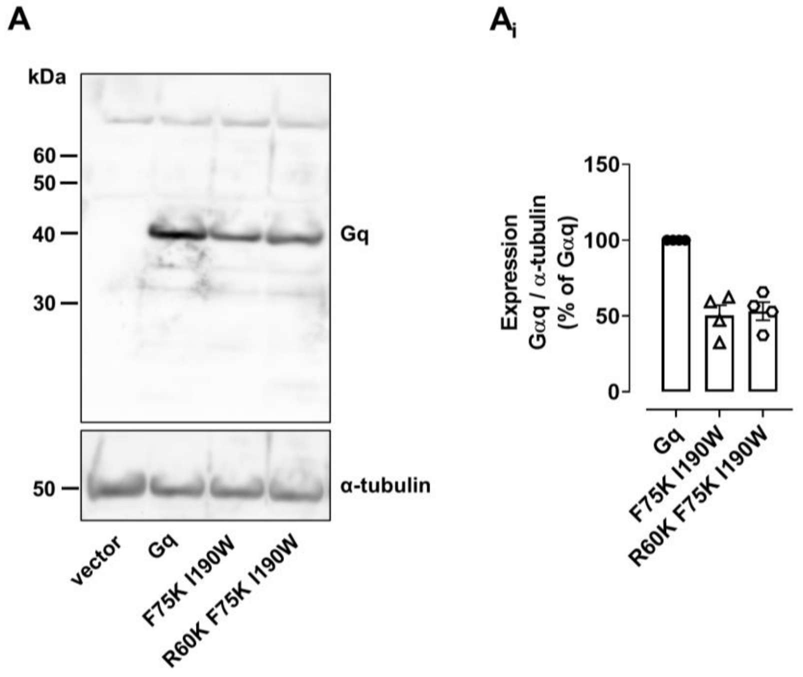

Figure S3. The FR-resistant triple and double mutant show similar cellular expression. A. Representative western blot showing expression levels of $G\alpha q^{WT}$ and mutant proteins detected in cellular lysates that were collected from CRISPR-Cas9 HEK-ΔGq/11 cells transiently expressing the indicated constructs and (**A$_i$**) quantification thereof. α-tubulin was used as the loading control. Three additional blots from independent transfections gave similar results. Images were spliced together to remove irrelevant samples. The third lane on this blot is identical to the right outermost lane in Figure S2. Columns represent means ± S.E. of four independent experiments.

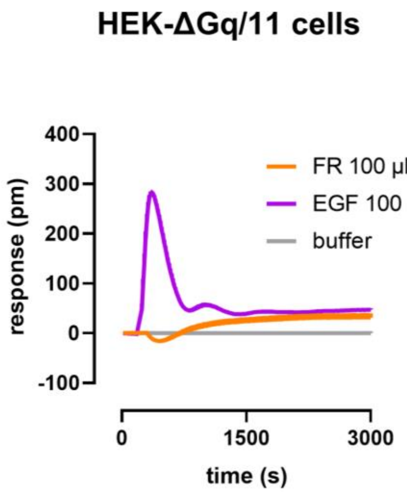

Figure S4. FR does not alter cell morphology in the absence of its biological target Gq. CRISPR-Cas9 HEK-ΔGq/11 cells respond to epidermal growth factor (EGF) with robust alteration of DMR profiles but show minimal cell morphology fluctuations when exposed to FR. Data shown are representative real-time recordings (mean + S.E., technical triplicates) of three independent experiments.

Table S1. Primers for site-directed mutagenesis.

Mutant	Forward primer (5′–3′)	Reverse primer (5′–3′)
Gq R60K	CAAGCAGATGAAGATCATCCACGGG	CCCGTGGATGATCTTCATCTGCTTG
Gq F75K	GCGCGGCAAAACCAAGCTGGTGTATCAG	CTGATACACCAGCTTGGTTTTGCCGCGC
Gq I190W	CAGGGATCTGGGAATACCCCTTTGACTTAC	GTAAGTCAAAGGGGTATTCCCAGATCCCTG

QuikChange forward and reverse primers employed to generate the indicated Gαq loss of function constructs are listed.
